# Identification and functional analysis of variants of *MYH6* gene promoter in isolated ventricular septal defects

**DOI:** 10.1186/s12920-022-01365-y

**Published:** 2022-10-08

**Authors:** Ji-Yang Zuo, Huan-Xin Chen, Zhi-Gang Liu, Qin Yang, Guo-Wei He

**Affiliations:** 1 Department of Cardiovascular Surgery, The Institute of Cardiovascular Diseases, TEDA International Cardiovascular Hospital, Tianjin University & Chinese Academy of Medical Sciences, No. 61, the 3rd Ave., Tianjin, 300457 China; 2 Clinical School of Cardiovascular Disease, Tianjin Medical University, Tianjin, China

**Keywords:** Ventricular septal defect, *MYH6*, Genetic, Variants, Congenital heart disease

## Abstract

**Background:**

Ventricular septal defect is the most common form of congenital heart diseases. *MYH6* gene has a critical effect on the growth and development of the heart but the variants in the promoter of *MYH6* is unknown.

**Patients and methods:**

In 604 of the subjects (311 isolated and sporadic ventricular septal defect patients and 293 healthy controls), DNA was extracted from blood samples and *MYH6* gene promoter region variants were analyzed by sequencing. Further functional verification was performed by cellular experiments using dual luciferase reporter gene analysis, electrophoretic mobility shift assays, and bioinformatics analysis.

**Results:**

Nine variants were identified in the *MYH6* gene promoter and two of those variants [g.4085G>C(rs1222539675) and g.4716G>A(rs377648095)] were only found in the ventricular septal defect patients. Cellular function experiments showed that these two variants reduced the transcriptional activity of the *MYH6* gene promoter (*p* < 0.001). Further analysis with online JASPAR database suggests that these variants may alter a set of putative transcription factor binding sites that possibly lead to changes in myosin subunit expression and ventricular septal defect formation.

**Conclusions:**

Our study for the first time identifies variants in the promoter region of the *MYH6* gene in Chinese patients with isolated and sporadic ventricular septal defect. These variants significantly reduced *MYH6* gene expression and affected transcription factor binding sites and therefore are pathogenic. The present study provides new insights in the role of the *MYH6* gene promoter region to better understand the genetic basis of VSD formation.

**Supplementary Information:**

The online version contains supplementary material available at 10.1186/s12920-022-01365-y.

## Introduction

Congenital heart defects (CHDs), characterized by structural and conduction abnormalities, are congenital anomalies that pose a serious threat to the health of children's lives and represent the most prevalent congenital birth defects, accounting for approximately one-third of all congenital defects [1, 2]. CHDs affects 9–18 out of every 1000 live births [3], and nearly 300,000 people die of congenital heart disease each year, 70% of whom are children under the age of one [4]. Advances in the treatment of CHDs have improved the survival rate of CHD patients and have largely led to an increase in its prevalence in older children and adults, who remain at high risk for continued disease burden after surgical repair, with the possibility of atrial arrhythmias, pulmonary hypertension, and other complications requiring repeat surgery, subsequently increasing the health expenditure and medical burden on society [5]. Therefore, it has become increasingly important to unravel the pathogenesis of CHDs. Previous studies have shown that genetic, epigenetic and environmental factors are causative factors of CHDs [3, 6, 7].

Ventricular septal defect (VSD) is the most common CHD, accounting for 30–40% of all CHDs [8, 9]. The incidence of this defect varies with age due to the varying sensitivity of screening techniques and the fact that many small malformations present at birth disappear shortly thereafter. The incidence of isolated VSDs by high-sensitivity color Doppler echocardiography screening has been reported to be 5.7% in preterm infants and 1.1–5.3% in full-term infants [10]. However, despite efforts to unravel the mechanisms of VSD formation, the exact mechanisms remain largely unknown.

*MYH6* (OMIM: 160710) is located on chromosome 14q11.2, and the protein it expresses is one of two myosin heavy chain isoforms expressed by the myocardium that mark cardiac progenitor cells and are involved in a variety of pluripotent cardiac cell lineages during embryonic development, being an important protein component in the formation of cardiac structure [11, 12]. Its potential impact on many forms of CHDs and cardiomyopathies, such as hypertrophic cardiomyopathy, dilated cardiomyopathy, sick sinus node syndrome, atrial septal defect, pulmonary atresia, and VSD has been reported [13–16]. Single nucleotide polymorphisms in *MYH6* have been found to be potentially associated with CHD [17, 18], whereas no reports have focused on variants in the promoter region of the *MYH6* gene causing CHD. Recent studies on gene promoter regions have shown that mutations and variations in promoter regions can alter the expression of genes, which may lead to related diseases [19–21]. In view of the fact that MYH6 protein is an important subunit of myosin during cardiac development, we hypothesized that variants in the *MYH6* promoter may change the expression of *MYH6* and may be involved in the formation of VSD. To test this hypothesis, we designed the present study to genetically and functionally analyze the DNA sequences of the *MYH6* promoter region in Chinese patients with isolated and sporadic VSD compared to healthy controls.

## Patients, materials and methods

### Study participants included

**Fig. 1:**
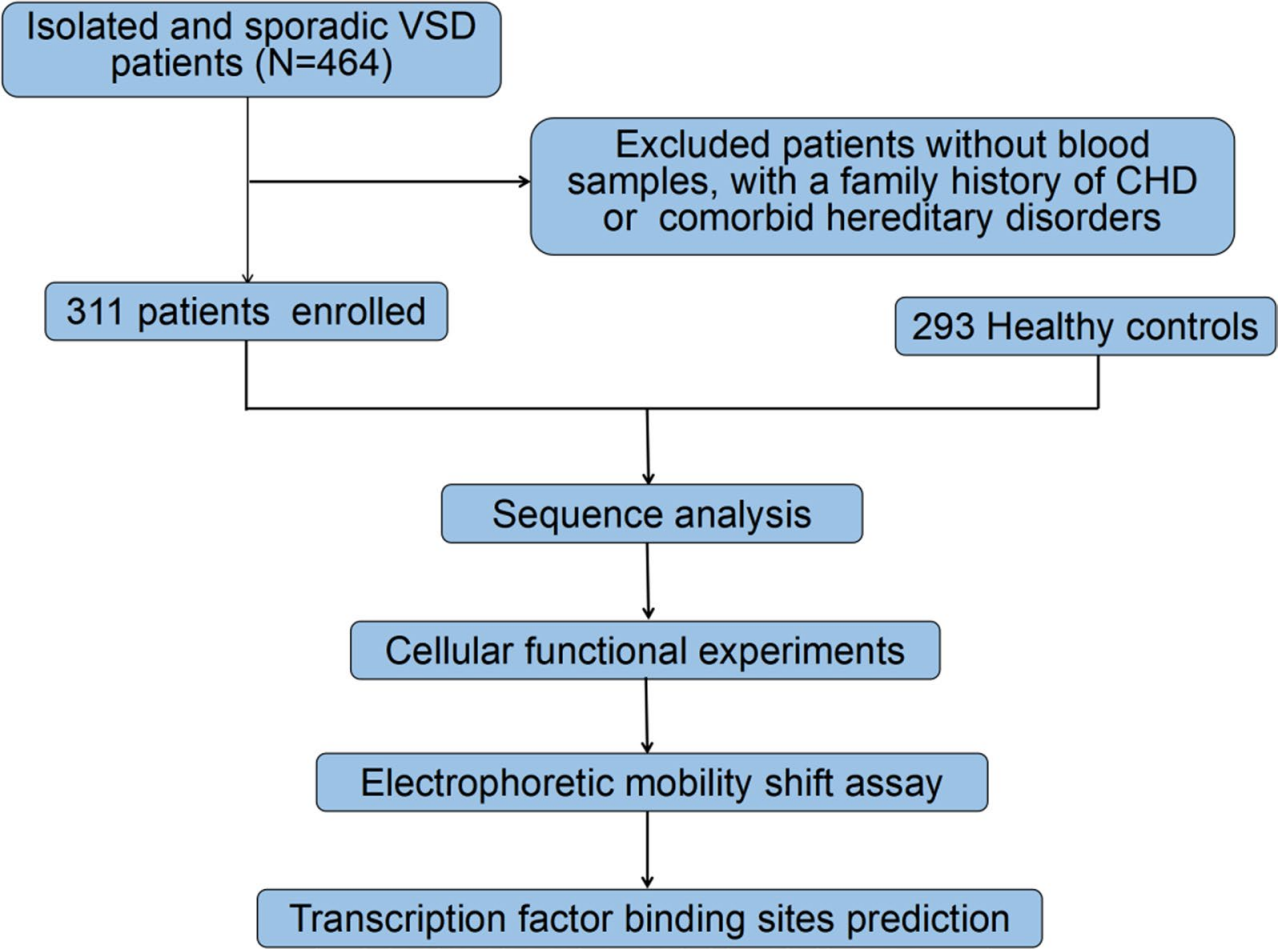
Study flow chart. A total of 604 subjects were recruited. DNA was extracted from blood samples. Sequence analysis and cellular function experiments were performed

This study enrolled 604 subjects. A total of 464 Chinese patients with isolated and sporadic VSD underwent repair surgery from January 2018 to August 2019 at the Department of Cardiovascular Surgery, TEDA International Cardiovascular Hospital, Tianjin, China. A total of 311 VSD patients (167 males and 144 females; age range: 1 month to 12 years) were enrolled in this study. The exclusion criteria were patients without blood samples, or with a family history of CHD, or with other comorbid hereditary disorders. The blood samples from those patients were collected. In addition, blood samples from 293 healthy controls (166 males and 127 females; age range: 1 month to 12 years) were also collected. The healthy controls were from heart disease screening program and body check program. All healthy subjects were confirmed free of CHD or other diseases and family history of genetic disorders by clinical screening including echocardiography. This work was performed in accordance with the principles of the Declaration of Helsinki and was approved by the Ethics Committee of TEDA International Cardiovascular Hospital. Written consent forms were informed and signed by the parents or guardians of the participants. The flow chart of the study is shown in Fig. 1.

### Sequence analysis

Fasting venous blood was collected and genomic DNA was extracted from peripheral leukocytes using the RelaxGene Blood DNA System kit (TianGen, Beijing, China). The sequence of the *MYH6* gene promoter was obtained from the GenBank database (NCBI, NG_023444.1) and was amplified from human genomic DNA. The design of the polymerase chain reaction (PCR) primers was based on the sequence of the human *MYH6* gene. The gene promoter is located upstream of the coding region starting at − 1500 bp to − 2000 bp, and the specific promoter sequence of *MYH6* was selected by BLAST in GenBank database (1638 bp, from − 1791 bp to − 154 bp to the transcription start site). Amplification conditions were: 95 °C for 5 min, followed by 35 cycles (denaturation at 95 °C for 30 s, annealing at 60 °C for 30 s, extension at 72 °C for 105 s), and a final extension at 72 °C for 10 min. The PCR products were sequenced in both directions for alignment and comparison with the wild-type *MYH6* gene promoter sequence. Primers for Sanger sequencing are shown in Table 1. DNA sequences were then compared to the wild-type *MYH6* gene promoter and regulatory variants, including single nucleotide polymorphisms (SNPs), were identified.

**Table 1:** List of primers used in this study

Primers name	Sequences	Location
PCR primers		
MYH6-F_1_	5'-GGGGCCTCGAGTAACCTAGA-3'	3209–3228
MYH6-R_1_	5'-CCCCCTGATTTGCCCAAGAA-3'	4846–4827
Sequencing primers		
MYH6-F_1_	5'-GGGGCCTCGAGTAACCTAGA-3'	3209–3228
MYH6-R_2_	5'-CCCCCTGATTTGCCCAAGAA-3'	4846–4827
MYH6-R_1_	5'-GACTTGACCGTGTCTGTGC-3'	4135–4154
Primers containing restriction sites		
MYH6-KpnI^a^	5'-**CGG**GGTACCGGGGCCTCGAGTAACCTAGA-3'	
MYH6-SacI^a^	5'-**AAA**AGTACTCCCCCTGATTTGCCCAAGAA-3'	
The double-stranded biotinylated oligonucleotides for the EMSA		
g.4085G>C-F	5'-CACACTTACCCACTCAAGCTTG(C/G)AGATTTCTTTTCTACTTTC-3'	
g.4085G>C-R	5'-GAAAGTAGAAAAGAAATCT(G/C)CAAGCTTGAGTGGGTAAGTGTG-3'	
g.4716G>A-F	5'-AAGACAGGACCTTCTCA(C/T)ACCGCCTCTCCCACCCT-3'	
g.4716G>A-R	5'-AGGGTGGGAGAGGCGGT(G/A)TGAGAAGGTCCTGTCTT-3'	
Nucleotide sequences for JASPAR prediction of TFBS		
g.4085G>C-F	5'-CACACTTACCCACTCAAGCTTG(C/G)AGATTTCTTTTCTACTTTC-3'	
g.4716G>A-F	5'-AAGACAGGACCTTCTCA(C/T)ACCGCCTCTCCCACCCT-3'	

PCR primers are designed based on the genomic DNA sequence of the *MYH6* gene (NG_023444.1). The transcription start site is at the position of 5,001 (+ 1)

EMSA, electrophoretic mobility shift assay; TFBS, transcription factor binding sites; F, forward; R, reverse

^a^Protective bases are presented in bold

### Plasmid constructs, cell culture, and transfection

To investigate the effect of variants on promoter activity, the wild-type and variant *MYH6* gene promoter fragments generated by PCR were inserted into the KpnI and SacI sites of pGL6-basic to construct gene expression vectors, which were verified by Sanger sequence analysis. The PCR primers with KpnI and SacI sites are listed in Table 1. The plasmids were extracted using Plasmid Mini Preparation Kit (Beyotime Biotechnology, Shanghai, China) according to the instructions. These expression vector plasmids were then transfected separately with pRL-SV40 (sea kidney luciferase reporter plasmid) in a 5:1 ratio into HEK-293 and HL-1 cells, which were cultured in Minimum Essential Medium supplemented with 10% fetal bovine serum. For cell transfection analysis, 1 × 10^6^ cells were transfected with 2.5 ng of plasmid DNA, and all groups were transfected together in six-well plates and subsequently examined for dual luciferase activity.

### Analysis with the dual-luciferase reporter assay

After 48 h of transfection, the cells were collected and lysed sufficiently to retain the lysate. Dual luciferase activity was measured using a dual luciferase reporter gene assay system (Beyotime Biotechnology, Shanghai, China). The empty vector pGL6-basic and blank were used as negative controls and pRL-SV40 was used as an internal control to correct for transfection efficiency. The results are presented in terms of the relative fold change of these construction vectors compared to the wild-type *MYH6* gene promoter in the expression vector. All experiments were repeated five times independently.

### Nuclear extracts preparation and electrophoretic mobility shift assay (EMSA)

To examine the effect of *MYH6* gene regulatory variants on transcription factor binding sites (TFBS), nuclear protein extracts of HEK-293 and HL-1 were prepared using the Nuclear and Cytoplasmic Protein Extraction Kit (Beyotime Biotechnology, Shanghai, China) according to the manufacturer's instructions to EMSA. Protein concentrations were quantified using the Enhanced BCA Protein Assay Kit (Beyotime Biotechnology, Shanghai, China). Biotinylated double-stranded oligonucleotides containing wild-type or variant DNA sequences in the *MYH6* gene promoter were used as probes. EMSA was performed using the chemiluminescent EMSA kit (Beyotime Biotechnology, Shanghai, China) using equal amounts of probes (0.25 pM) and nuclear extracts (2.0 μg) according to the manufacturer's protocol. The EMSA oligonucleotide sequences are shown in Table 1.

### TFBS and interacting proteins prediction

JASPAR (https://jaspar.genereg.net/) [22] (version 9) was used to predict the possible binding transcription factors (TFs) [23]. To further investigate whether *MYH6* gene promoter variants disrupt or generate TFBS, we used JASPAR to generate a list of all potentially affected binding sites through the identified variants with a relative profile score threshold set at 85%. The wild-type and variant nucleotide sequences are shown in Table 1. STRING (https://cn.string-db.org/) is an online searchable database of known protein–protein interaction (PPI) [24]. By using the protein names provided by the user, the interacting proteins can be more accurately predicted. We used STING to find proteins that may be affected by mutations in the *MHY6* gene with setting the organism to "Homo sapiens" and setting the minimum required interaction score ≥ 0.700. We then imported the resulting PPI maps into Cytoscape 3.9.1 software for network topology analysis [25].

### Statistical analysis

The quantitative data were expressed as mean ± SD and compared by one-way analysis of variance. The frequency of DNA sequence variants was compared with that of VSD patients and healthy controls using the Pearson’s chi-square. All statistical analyses were performed with SPSS 25.0 software. *p* < 0.05 was considered statistically significant.

## Results

### Variants found in VSD patients by DNA sequence

**Fig. 2:**
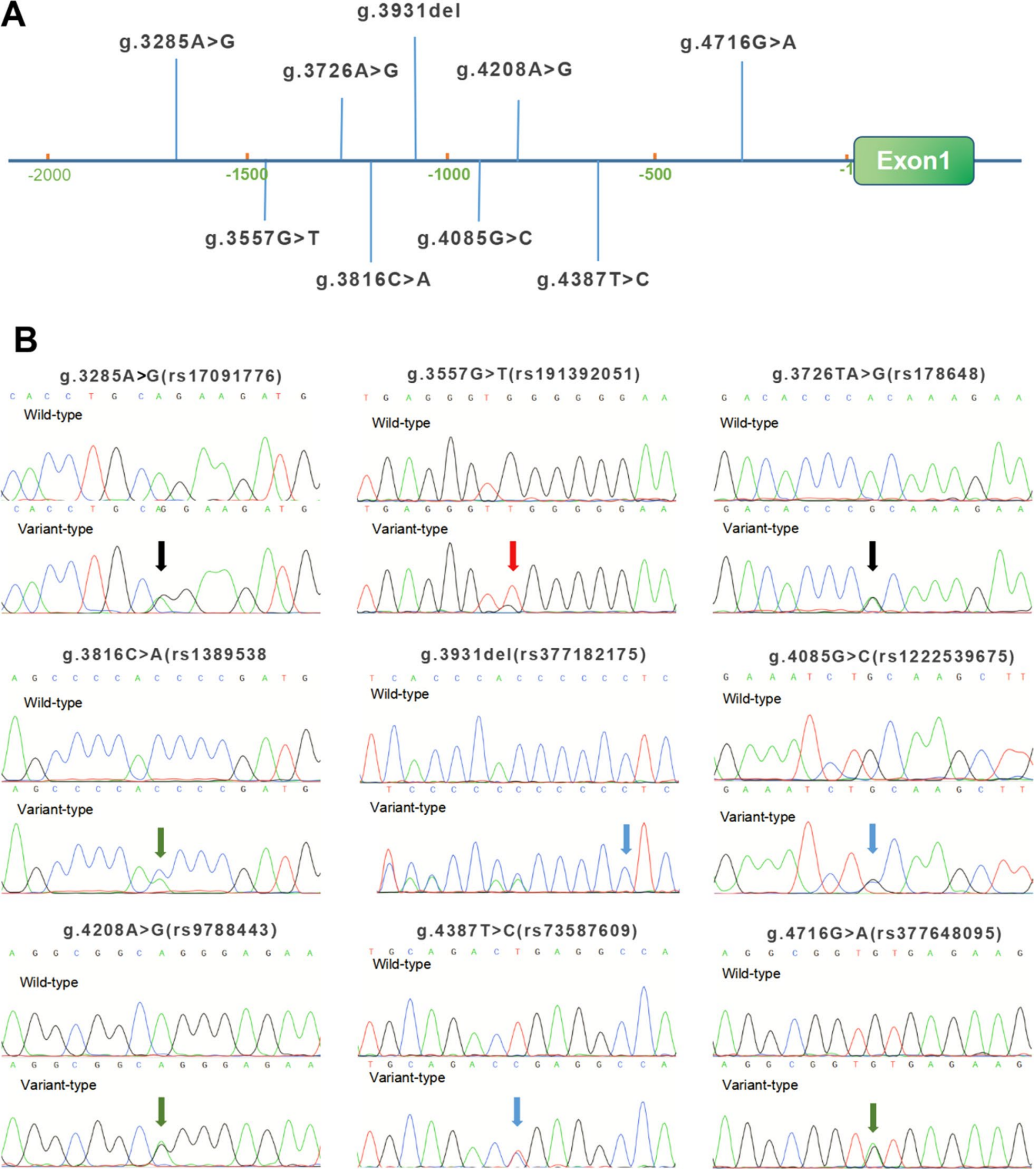
Locations and sequencing chromatograms of *MYH6* gene promoter variants. **A** Genetic variants are named according to the genomic DNA sequence of the human *MYH6* gene (Genbank accession number NG_023444.1). The transcription start site is at position 5,001 in the first exon. **B** Sequencing chromatograms of all variants found in VSD patients and controls (the sequencing peaks are reverse sequencing). Top panels show wild-type and bottom panels show variant-type, marked with arrows. VSD, ventricular septal defect

In the 604 Chinese subjects recruited for this study, nine variants were identified by Sanger sequencing. All of these variants are summarized in the Table 2. The locations are shown in Fig. 2A and their sequencing chromatograms are shown in Fig. 2B. Among these 9 variants, 2 single nucleotide variants [g.4085G>C(rs1222539675) and g.4716G>A(rs377648095)] were found only in VSD patients but the other 7 variants were found in both VSD patients and healthy controls (Table 2). Importantly, in the NCBI SNP database, the above two variants only found in the VSD patients had allele frequencies < 0.0001 [g.4085G>C(rs1222539675) variant rate C = 0.000019 and g.4716G>A(rs377648095) variant rate T = 0.000011], suggesting the possible pathogenic role of these variants. Therefore, these two variants were further studied in cellular function experiments to reveal the pathological role. In contrast, the other seven variants found in both VSD patients and controls were excluded from further studies.

**Table 2:** Variants within the *MYH6* gene promoter in patients with VSD

Variations	Position^a^	Genotypes	VSD^b^	Controls^b^	Frequency^c^	*P* value
**g.4085G>C(rs1222539675)**	− **915**	**G>C**	**1**	**0**	**C = 0.000019**	**–**
**g.4716G>A(rs377648095)**	− **284**	**G>A**	**1**	**0**	**A = 0.000011**	**–**
g.3285A>G(rs17091776)	− 1715	A>G	56	42	G = 0.1048	0.221
g.3557G>T(rs191392051)	− 1443	G>T	18	10	T = 0.0038	0.165
g.3726A>G(rs178648)	− 1244	A>G	7	2	G = 0.0046	0.178
g.3816C>A(rs138953808)	− 1184	C>A	8	9	A = 0.0224	0.711
g.3931del(rs377182175)	− 1069	delC	5	4	delC = 0.0014	1.0
g.4208A>G(rs9788443)	− 972	A>G	63	45	G = 0.1767	0.116
g.4387T>C(rs73587609)	− 613	T>C	62	46	C = 0.0477	0.175

^a^Variants are located upstream (−) to the transcription start site at the position of 5,001 (+ 1) of the *MYH6* gene (NG_023444.1)

^b^Allele frequency in groups. VSD, ventricular septal defect

^c^The allele frequency was obtained from NCBI dbSNP database

### Regulatory variants alter the activity of the MYH6 gene promoter

**Fig. 3:**
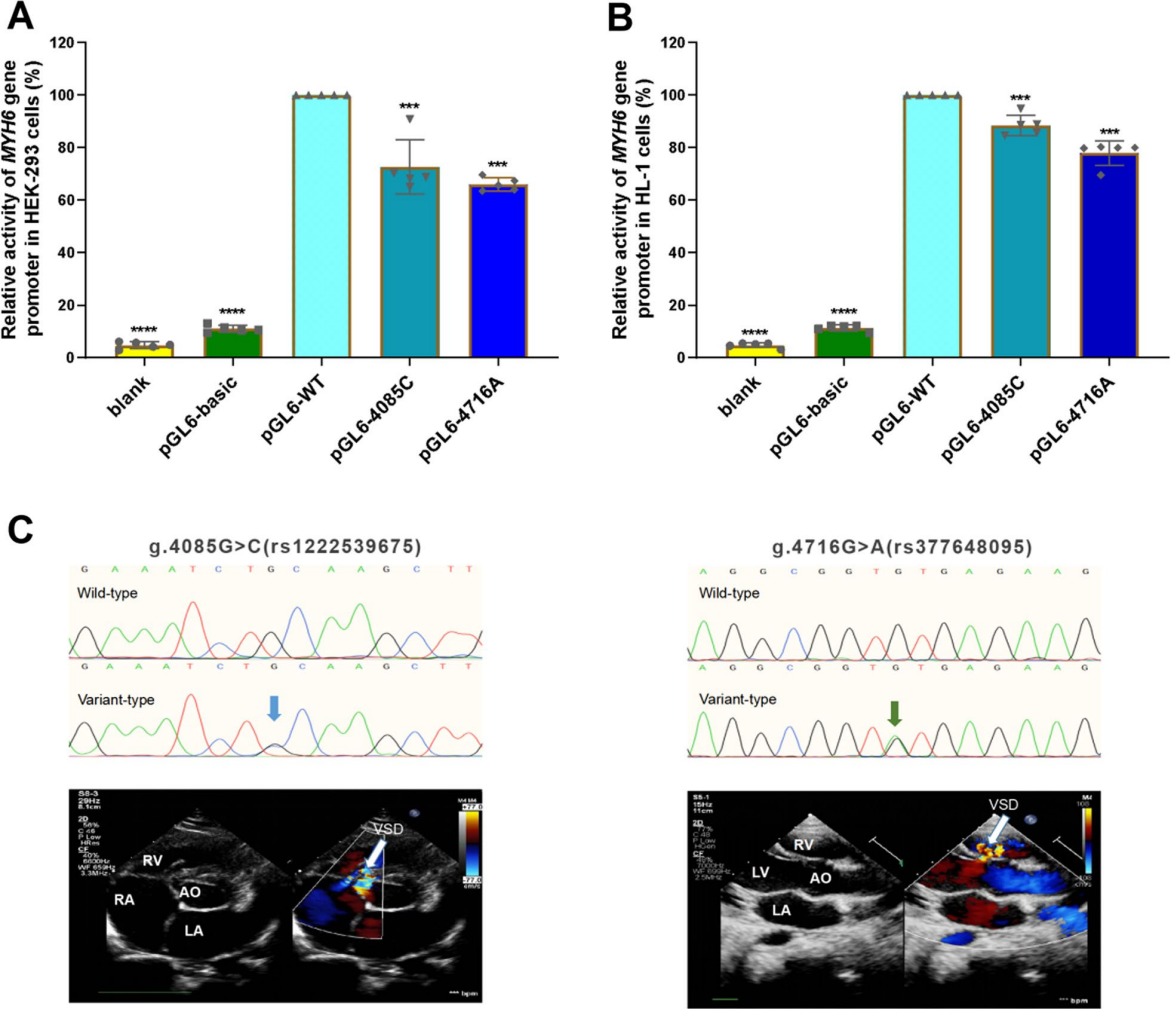
Results of dual luciferase reporter gene analysis. **A** Relative transcriptional activity of wild-type and variants of *MYH6* gene promoters in HEK-293 cells. The transcriptional activity of the wild-type *MYH6* gene promoter was set as 100%. The relative activities of *MYH6* gene promoters were calculated. Quantitative data are expressed as mean ± SD and are based on five independent experiments (n = 5, ***P < 0.001, ****P < 0.0001). **B** Relative transcriptional activity of wild-type and variants of *MYH6* gene promoters in HL-1 cells.. The transcriptional activity of the wild-type *MYH6* gene promoter was set as 100%. The relative activities of *MYH6* gene promoters were calculated. Quantitative data are expressed as mean ± SD and are based on five independent experiments (n = 5, ***P < 0.001, ****P < 0.0001). **C** Color Doppler echocardiography shows VSD (arrow) in patients with variants of g.4085G>C (rs1222539675) and g.4716G>A (rs377648095). AO, aorta; LA, left atrium; RA, right atrium; RV, right ventricle; LV, left ventricle; VSD, ventricular septal defect

In order to examine the effect of the variants in the *MYH6* gene promoter on promoter activity, the wild-type and variant *MYH6* gene promoters were cloned into the firefly luciferase reporter plasmid (pGL6-basic) reporter vector to generate constructed expression vectors, including blank, empty pGL6-basic (negative control), pGL6-WT (wild-type *MYH6* gene promoter), pGL6-4085C (g.4085G>C) and pGL6-4716A (g.4716G>A), respectively. These constructed vectors were then transfected into HEK-293 and HL-1 cells and dual luciferase activity was measured. The transcriptional activity of the blank and negative control groups was much lower than the other three groups, nearly 0. The two variants, g.4085G>C (rs1222539675) and g.4716G>A (rs377648095) found in VSD patients, significantly reduced the gene promoter expression activity and decreased luciferase expression compared to the wild-type *MYH6* gene promoter (*p* < 0.001) in HEK-293 (Fig. 3A) and HL-1 cells (Fig. 3B). The color Doppler echocardiography of the patients with these two variants is shown in Fig. 3C.

### EMSA analysis

**Fig. 4:**
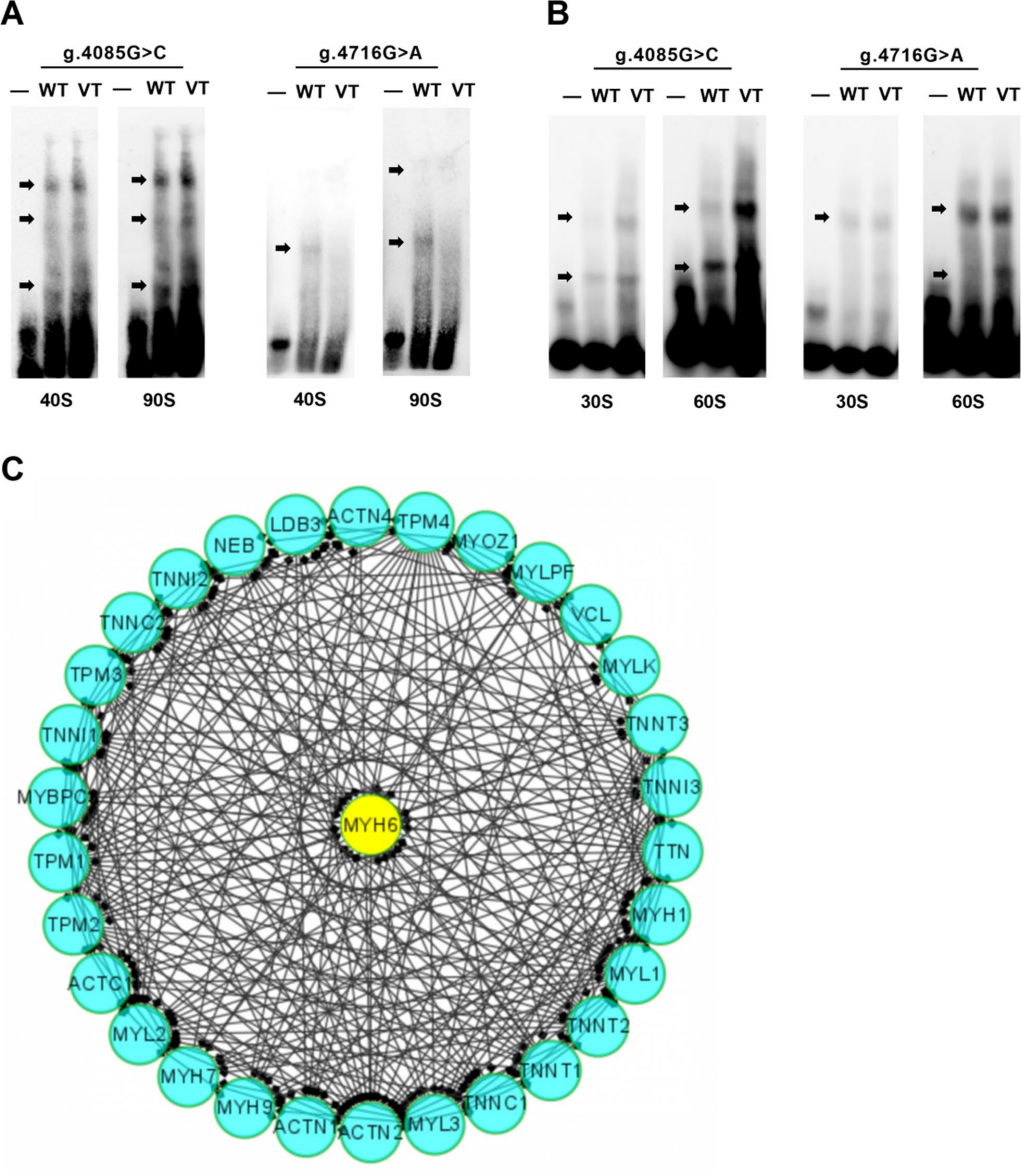
Cellular function experiments.** A** At different times (40 s and 90 s), two variants (g.4085G>C and g.4716G>A) were seen to create or abolish the binding of TFs in HEK-293 cells. **B** At different times (30 s and 60 s), two variants (g.4085G>C and g.4716G>A) were seen to create or abolish the binding of TFs in HL-1 cells. **C** The protein–protein interaction showing that MYH6 protein interacts with a number of proteins that playimportant role in the cardiac formation including TNNI1, MYBPC3, MYL2, ACTC1, ACTN2, MYL1, TNNT2, TPM1, and TNNI3, exec. EMSA, electrophoretic mobility shift assay; WT, wild-type; VT, variant-type

To experimentally investigate whether regulatory variants affect TF binding, EMSA was tested using wild-type or variant biotin-labeled probes. Regulatory variants found only in VSD patients were examined. The biotinylated oligonucleotides of EMSA are shown in Table 1. As shown in Fig. 4A, the variant g.4085G>C enhances the binding of TFs. In contrast, variant g.4716G>A significantly abolished the binding of TFs and created binding sites for new TFs in HEK-293 cells. Similarly, in HL-1 cells (Fig. 4B), the variant g.4085G>C and variant g.4716G>A enhance the binding of TFs in HL-1 cells (The original figures are provided in Additional file 1: Fig. S1). The brightness of the band may reflect the binding ability of TFs [26].

### The TFBS affected by regulatory variants and the PPI of MYH6

**Fig. 5:**
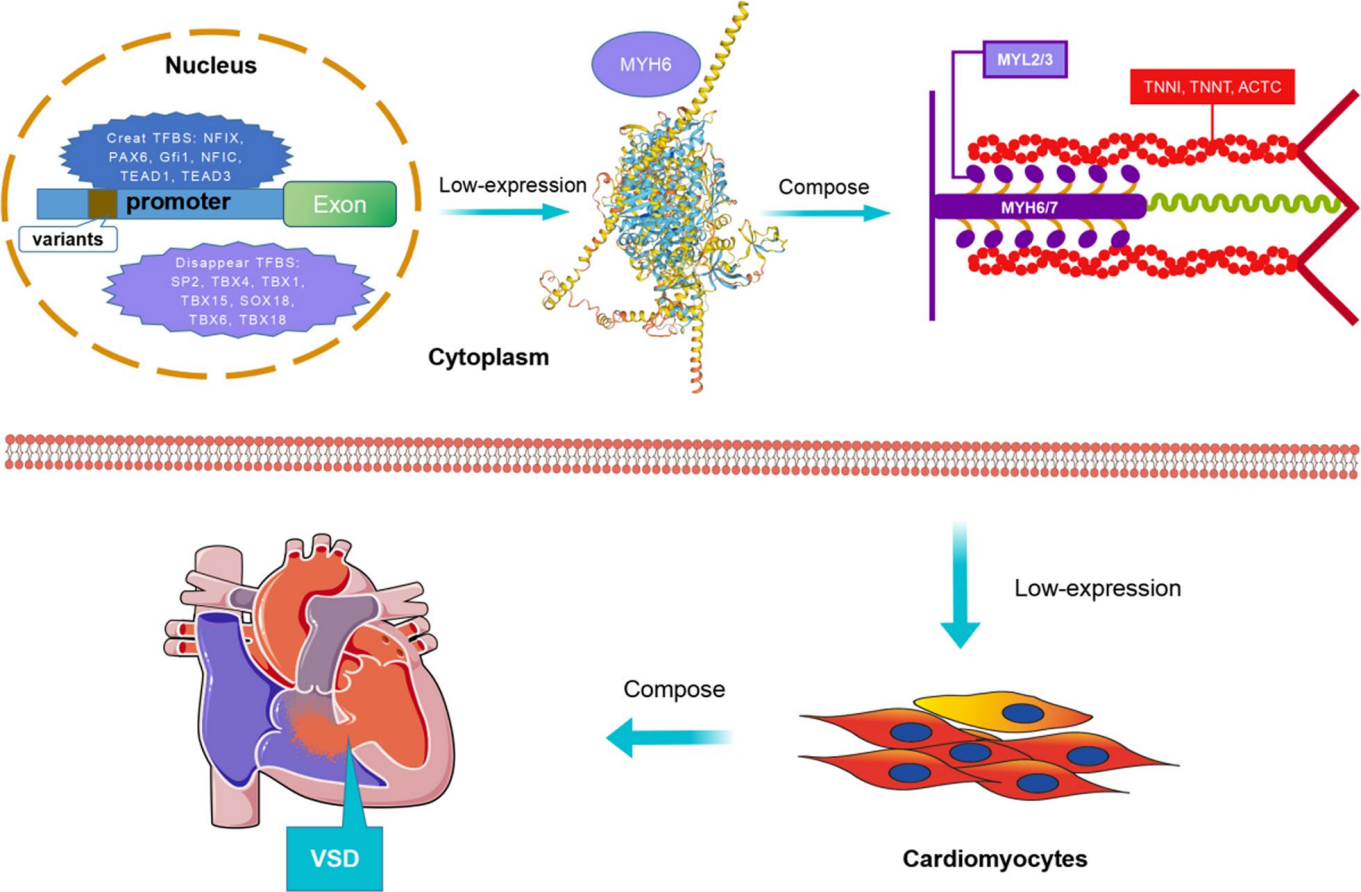
The schema describing the role of variants of *MYH6* gene promoter. Schema describes the role of variants in the promoter region of the *MYH6* gene identified from this study in connection with the analysis of the JASPAR database and findings from previous studies. Briefly, the variants in the *MYH6* gene promoter identified in this study may modify the cluster of TFBS, resulting in altered gene expression. Low-expression of MYH6 is associated with attenuated myosin heavy chain formation that is directly related to the development of VSD. In addition, variants in *MYH6* promoter affect the binding of TFs such as TBX1, TBX4, TBX6, TBX15 and TBX18, possibly resulting in formation of VSD. TF, transcription factor; TFBS, transcription factor binding sites; VSD, ventricular septal defect

Based on the JASPAR core TF database, we predicted potential TFBS that may be disrupted or generated by variants in the *MYH6* gene promoter. The results showed that various TFs play important roles in cardiogenesis, such as *E2F1*, *GATA4*, *NKX2-5*, *GATA2*, *GATA1*, *GATA6*, etc. As shown in the Table 3, variant g.4085G>C may generate binding sites for *NFIX*, *PAX6*, *Gfi1* and *NFIC* whereas variant g.4716G>A may produce binding sites for *TEAD1* and *TEAD3* and disrupt binding sites for *SP2*, *TBX4*, *TBX1*, *TBX15*, *SOX18*, *TBX6*, and *TBX18*. Based on these analyses by using the JASPAR database, cellular function experiments, and previous studies, we established a schema to describe the possible effects of variants in the promoter region of the *MYH6* gene on the development of VSD as shown in Fig. 5. The construction of PPI networks using STRING was analyzed by Cytoscape 3.9.1, and a total of 31 nodes and 300 edges were obtained (Fig. 4C). The nodes of the network graph represent the protein names and the edges represent their interactions. The top ten proteins that interacted most strongly with MYH6 protein were TNNI1, MYBPC3, MYL2, ACTC1, ACTN2, MYL1, TNNT2, TPM1, and TNNI3.

**Table 3:** TFBS affected by variants (predicted by the JASPAR database)

	Wild^a^	Variant^b^	Disrupt	Create
g.4085G>C	NKX2-5, NKX3-2, ZNF354C, FOXC1, NKX3-1, GATA3, GATA2, GATA1, NR2E3, NFAT5, EN2, NKX2-8, RHOXF1, HIC2, ISL2, BARHL1, MSANTD3, HOXB6, DLX1, DlX4, GATA6, DLX6, DlX3, BARX1, FOXP3, DlX2, NKX2-2, NKX2-4, SOX18, ZNF784, ZIM3	NKX2-5, NKX3-2, ZNF354C, FOXC1, NKX3-1, GATA3, GATA2, GFI1, GATA1, PAX6, NR2E3, NFAT5, NFIC, EN2, NKX2-8, RHOXF1, HIC2, NFIX, ISL2, BARHL1, MSANTD3, HOXB6, DLX1, DlX4, GATA6, DLX6, DlX3, BARX1, FOXP3, DlX2, NKX2-2, NKX2-4, SOX18, ZNF784, ZIM3	–	NFIX, PAX6, Gfi1, NFIC
g.4716G>A	E2F1, KLF4, SP1, SPI1, KLF5, MEIS1, E2F6, EGR1, GATA4, KLF1, E2F4, SP2, MGA, MEIS3, TBX4, TBX5, TBX1, TBX15, SP8, SP3, KLF14, NR2C2, NR4A1, ZNF75D, SOX18, WT1, TBX6, TBX18, ZNF281, PRDM9, SP9, TBX3	E2F1, KLF4, SP1, SPI1, KLF5, MEIS1, E2F6, EGR1, GATA4, KLF1, E2F4, MGA, MEIS3, TBX5, SP8, SP3, KLF14, NR2C2, NR4A1, ZNF75D, WT1, ZNF281, PRDM9, SP9, TBX3, TEAD1, TEAD3	SP2, SOX18, TBX1, TBX4, TBX6, TBX15, TBX18	TEAD1, TEAD3

TFBS, transcription factor binding sites

^a^Wild-type possessed TFBS

^b^Variant-type possessed TFBS

## Discussion

The present study focused on variants in the *MYH6* gene promoter in the Han Chinese patients with isolated and sporadic VSD and has for the first time found that (1) among the 9 regulatory variants identified within the promoter region of the *MYH6* gene in the 604 human subjects, two variants [g.4085G>C(rs1222539675) and g.4716G>A(rs377648095)] were only detected in VSD patients in the study population; (2) these two variants cause cellular functional changes demonstrated in dual luciferase activity and EMSA; (3) These variants may cause alterations in TFBS and also affect proteins that interact with the MYH6 protein, resulting in altered function and ultimately leading to VSD.

*MYH6* gene encodes the α heavy chain subunit of cardiac myosin [27], which is expressed in the heart from fetal life to adulthood and is actively involved in muscle force production and contraction [11, 12]. Altered transcriptional activity of the *MYH6* gene has been reported to affect the formation of myocardial protofibrils in animal experiments, resulting in the inability to form heart valves and the formation of heart trabeculae or even death [28, 29]. Recent studies have shown that genetic polymorphisms in *MYH6* are associated with risk of VSD [17, 18, 30]. Further, there is growing evidence that variants in the gene promoter play a key role in cardiac transcriptional regulation [19–21, 31, 32]. Based on the STRING database and a previous study [33], we found that the major proteins interacting with MYH6 protein are basically involved in myocardial development. Alterations in MYH6 protein expression may affect the interaction with other proteins that may have important implications for myocardial development. Therefore, the low-expression of MYH6 protein may lead to defects in myocardial formation.

It has been reported that variants in the *MYH6* gene may affect the formation of the myosin complex [34, 35]. However, mutations or variants in the promoter region of the *MYH6* gene have not been reported. In our study population (n = 604), *MYH6* gene regulatory variants were identified in VSD patients with a frequency of 0.64% (2/311) and the frequency in the total study population was 0.33% (2/604). Further, in this study we demonstrated that these two variants in the promoter region of the *MYH6* gene significantly lowered the transcriptional activity of *MYH6*, demonstrated by the changes induced by these variants in dual fluorescein reporter analysis. As well known, changes of the activity of *MYH6* affect the formation of myosin heavy chains [27].

In the present study, EMSA experiments showed that two variants [g.4085G>C (rs1222539675) and g.4716G>A (rs377648095)] only found in VSD patients had altered binding affinity to TFs. As reported, changes of binding affinity to TFs are critical in the transcriptional regulation of gene expression that leads to disease [19–21, 36]. Therefore, our study suggests that variants in the *MYH6* gene promoter may alter the TFBS cluster bound in the promoter, thereby reducing *MYH6* transcript levels that may be involved in the development of VSD through specific pathways.

Based on the JASPAR database analysis, two variants [g.4085G>C (rs1222539675) and g.4716G>A (rs377648095)] have been predicted to have TFBS generation or disappearance. Among the TFBS derived from the analysis, it has been demonstrated that *PAX6* inhibited the differentiation of cardiac fibroblasts [37]. In contrast, most of the TFBS that existed in the wild type but were deleted in the presence of the variants play essential roles in cardiovascular development, such as *SP2* and *SOX18* [38, 39]. T-box family TFs play essential roles in the development of the embryonic heart [40, 41]. It was predicted that variant g.4716G>A may attenuate the bindings of *TBX1*, *TBX4*, *TBX6*, *TBX15* and *TBX18* to the promoter region of the *MYH6* gene. In fact, these genes are differentially expressed at various stages of heart development, participating in the formation of the heart [42–46]. In summary, the variants in the promoter of *MYH6* gene identified in this study may modify the TFBS clusters in the promoter and interfere with the formation of transcriptional regulatory complexes with related TFs, leading to the formation of VSD. Figure 5 is a schema that illustrates these results.

As to the variants other than the two variants that were only found in the VSD cases (Table 2), although some of them have low frequency in the NCBI dbSNP database (< 1%), whether they may also affect the expression needs to be further studied. In this study, due to the fact that they were also found in the controls, we excluded them from further cellular experiments.

### Limitation

Although this study investigated samples from VSD patients and control subjects and the variants found had further functional tests at cellular level to reveal the potential pathogenic role, further animal experiments are needed to confirm the role of these variants in the development of VSD. This will be considered in our future studies.

## Conclusions

Our study identified nine variants in the promoter region of the *MYH6* gene in Chinese patients with isolated and sporadic VSD, two of which were found only in VSD patients. Furthermore, cellular function experiments, EMSA and bioinformatics analyses showed that these variants remarkably altered the expression of the *MYH6* gene and affected the binding of TFs that may contribute to the development of VSD. Therefore, the present study may provide new insights into the role of gene promoter regions to better understand the genetic basis of VSD formulation and facilitate further studies on the mechanisms of VSD formation.

## Supplementary Information

**Additional file 1. Fig. S1:** The original images of the EMSA of the two variants (g.4085G>C and g.4716G>A) with nuclear proteins from HEK-293 cells and HL-1 cells. EMSA, electrophoretic mobility shift assay; WT, wild-type; VT, variant-type.

## Data Availability

The authors are not able to share the clinical data due to full anonymisation of the data is very difficult. The reference gene requence in our study was based on chromosome 14 (https://www.ncbi.nlm.nih.gov/nuccore/301898188). All sequencing data used to support the findings of this study are available from https://doi.org/10.6084/m9.figshare.20101322.v1 and the corresponding author upon request.

## Abbreviations

CHD: Congenital heart defect
VSD: Ventricular septal defect
PCR: Polymerase chain reaction
SNP: Single nucleotide polymorphism
TFBS: Transcription factor binding sites
TF: Transcription factor
EMSA: Electrophoretic mobility shift assay
PPI: Protein–protein interaction
WT: Wild-type
VT: Variant-type
AO: Aorta
LA: Left atrium
RA: Right atrium
RV: Right ventricle
LV: Left ventricle
