# Supplementary material for: Identification and functional analysis of variants of MYH6 gene promoter in isolated ventricular septal defects
Source: BMC Med Genomics. 2022 Oct 8;15:213. doi: 10.1186/s12920-022-01365-y (PMC9548206; doi:10.1186/s12920-022-01365-y)
Supplement: Supplementary file 1 — Additional file 1. Fig. S1: The original images of the EMSA of the two variants (g.4085G>C and g.4716G>A) with nuclear proteins from HEK-293 cells and HL-1 cells. EMSA, electrophoretic mobility shift assay; WT, wild-type; VT, variant-type. [file 12920_2022_1365_MOESM1_ESM.docx]

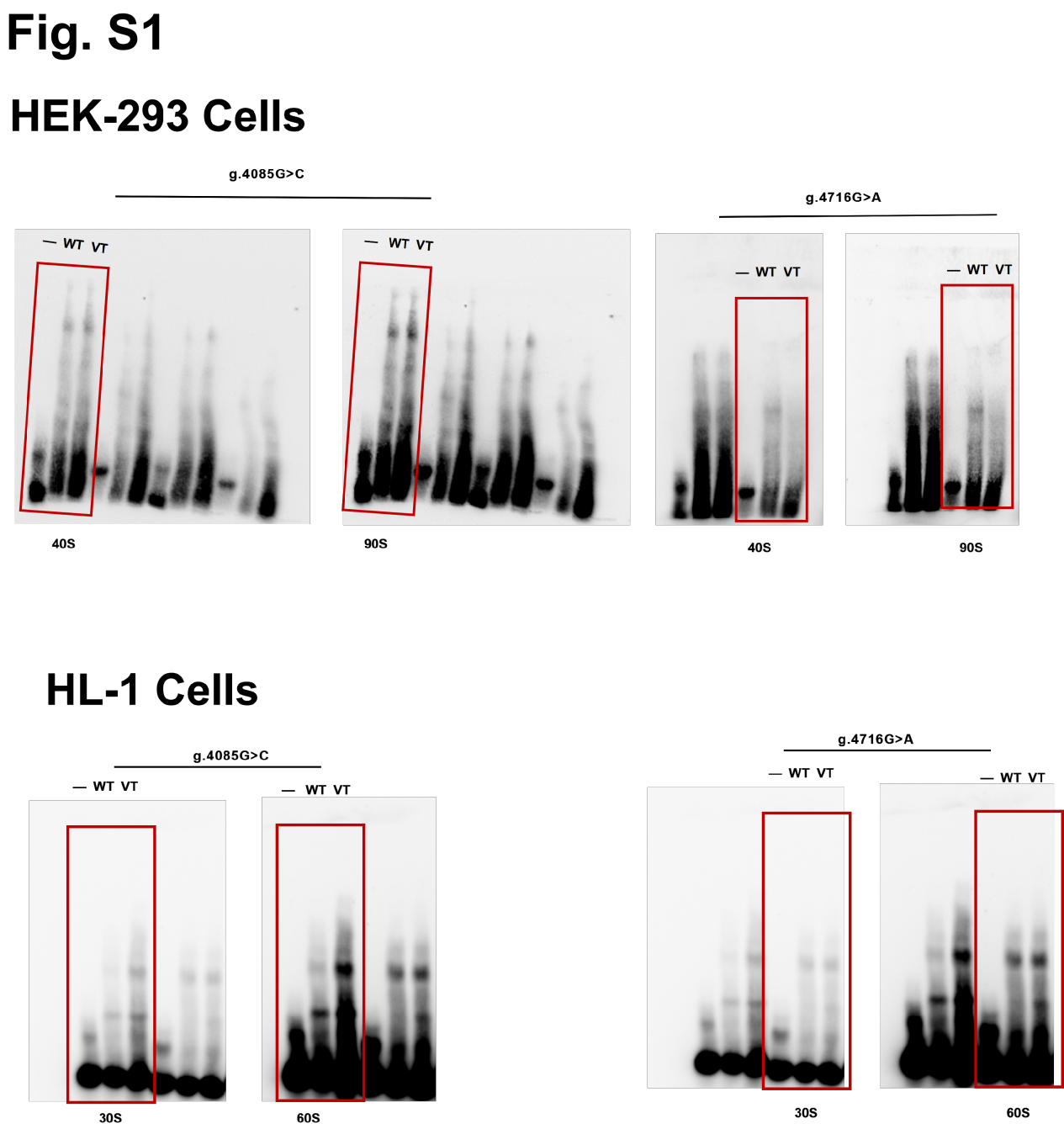


**Fig. S1.**  **Original images of the EMSA of the two variants in HEK-293 and HL-1 cells.**  The original images of the EMSA of the two variants (g.4085G>C and g.4716G>A) with nuclear proteins from HEK-293 cells and HL-1 cells. EMSA, electrophoretic mobility shift assay; WT, wild-type; VT, variant-type.
